# *BRCA2* reversion mutations as a resistance mechanism to olaparib in patients with *BRCA2*-mutated mCRPC: a case series

**DOI:** 10.1186/s13053-026-00346-x

**Published:** 2026-07-28

**Authors:** Kohji Takemura, Ayumi Abe, Kazushige Sakaguchi, Shinji Urakami, Masami Arai, Yuji Miura

**Affiliations:** 1 Department of Medical Oncology, Toranomon Hospital, Tokyo, Japan; 2 Department of Advanced Medical Development, The Cancer Institute Hospital of Japanese Foundation for Cancer Research, Tokyo, Japan; 3 Clinical Genetics Center, Toranomon Hospital, Tokyo, Japan; 4 Department of Clinical Genetics, Graduate School of Medicine, Juntendo University, Tokyo, Japan; 5 Department of Urology, Toranomon Hospital, Tokyo, Japan; 6 Department of Medical Oncology, The Cancer Institute Hospital of Japanese Foundation for Cancer Research, 3-8-31, Ariake, Koto-ku, Tokyo, 135-8550 Japan

**Keywords:** *BRCA2*, Reversion mutation, PARP inhibitor resistance, Olaparib, Liquid biopsy

## Abstract

**Background:**

Prostate cancer with *BRCA2* mutations has shown sensitivity to poly (ADP-ribose) polymerase (PARP) inhibitors such as olaparib. However, acquired resistance remains a clinical concern. One potential mechanism of resistance is the emergence of reversion mutations that restore the function of BRCA2. Although this mechanism has been observed in other tumor types, only a limited number of clinical reports have described this phenomenon in prostate cancer. This case series highlights three patients in whom *BRCA2* reversion mutations were detected following disease progression on olaparib.

**Case presentation:**

We present three male patients diagnosed with metastatic castration-resistant prostate cancer, each carrying a germline *BRCA2* mutation. All patients initially responded to olaparib, with a reduction in prostate-specific antigen levels and radiographic disease control. However, after approximately one year of treatment, each patient developed disease progression. Circulating tumor DNA analysis using a commercial liquid biopsy platform was performed after progression. In all cases, additional *BRCA2* mutations were detected, including multiple variants predicted to restore the disrupted open reading frame of the *BRCA2* gene. These findings were consistent with reversion mutations potentially restoring BRCA2 protein function and thereby conferring resistance to the PARP inhibitor. One patient additionally developed neuroendocrine differentiation during disease progression, highlighting the complexity of resistance evolution.

**Conclusions:**

This case series provides clinical evidence that *BRCA2* reversion mutations may underlie acquired resistance to olaparib in prostate cancer. These observations support the utility of liquid biopsy for detecting molecular mechanisms of resistance and suggest that reversion mutations may serve as a biomarker of treatment failure. Understanding such resistance mechanisms is essential for optimizing treatment sequencing and developing novel therapeutic strategies in *BRCA2*-mutated prostate cancer.

**Supplementary Information:**

The online version contains supplementary material available at https://doi.org/10.1186/s13053-026-00346-x.

## Background

*BRCA1/2* mutations are key mechanisms of carcinogenesis in various cancers, including breast, ovarian, pancreatic, and prostate cancers. *BRCA* genes function as tumor suppressors, and according to the two-hit hypothesis, their inactivation often involves a combination of germline mutation and loss of heterozygosity [1].

Therapies based on synthetic lethality have been developed for *BRCA*-mutated cancers, and PARP inhibitors have shown particular efficacy [2]. However, disease progression due to acquired resistance to PARP inhibitors remains a clinical challenge. One notable resistance mechanism is reversion mutation, where additional mutations restore the open reading frame and functional activity of BRCA1/2 proteins [3, 4].

Here, we report three cases of acquired resistance to the PARP inhibitor olaparib, potentially due to *BRCA2* reversion mutations identified via circulating tumor DNA (ctDNA) analysis in patients with germline *BRCA2*-mutated metastatic castration-resistant prostate cancer (mCRPC).

## Case 1

A 57-year-old man was diagnosed with metastatic hormone-sensitive prostate cancer (mHSPC) involving pelvic bone metastasis. His initial PSA level was 13.43 ng/mL, and the prostate biopsy showed Gleason score 4 + 4 = 8 (ISUP Grade Group 4). His family history included colorectal cancer in his father and gastric cancer in his maternal aunt (Supplement 1).

He received degarelix, bicalutamide, and intensity-modulated radiation therapy (IMRT) to the prostate (78 Gy/39 fractions). His PSA decreased to a nadir of 0.01 ng/mL but increased again 3.5 years later, indicating progression to mCRPC. Subsequent treatments included: enzalutamide for 2 years, abiraterone acetate plus prednisolone for 2.5 years, docetaxel plus prednisolone (3 cycles), and Radium-223.

Despite these treatments, he showed PSA and radiographic progression, including newly developed liver metastases.

Germline *BRCA2* mutation testing using BRACAnalysis^®^ revealed a c.281del (p.Pro94Leufs*2) mutation. This single-nucleotide deletion caused a frameshift and a premature stop codon at position 94, truncating the BRCA2 protein and eliminating its functional domains.

**Fig. 1:**
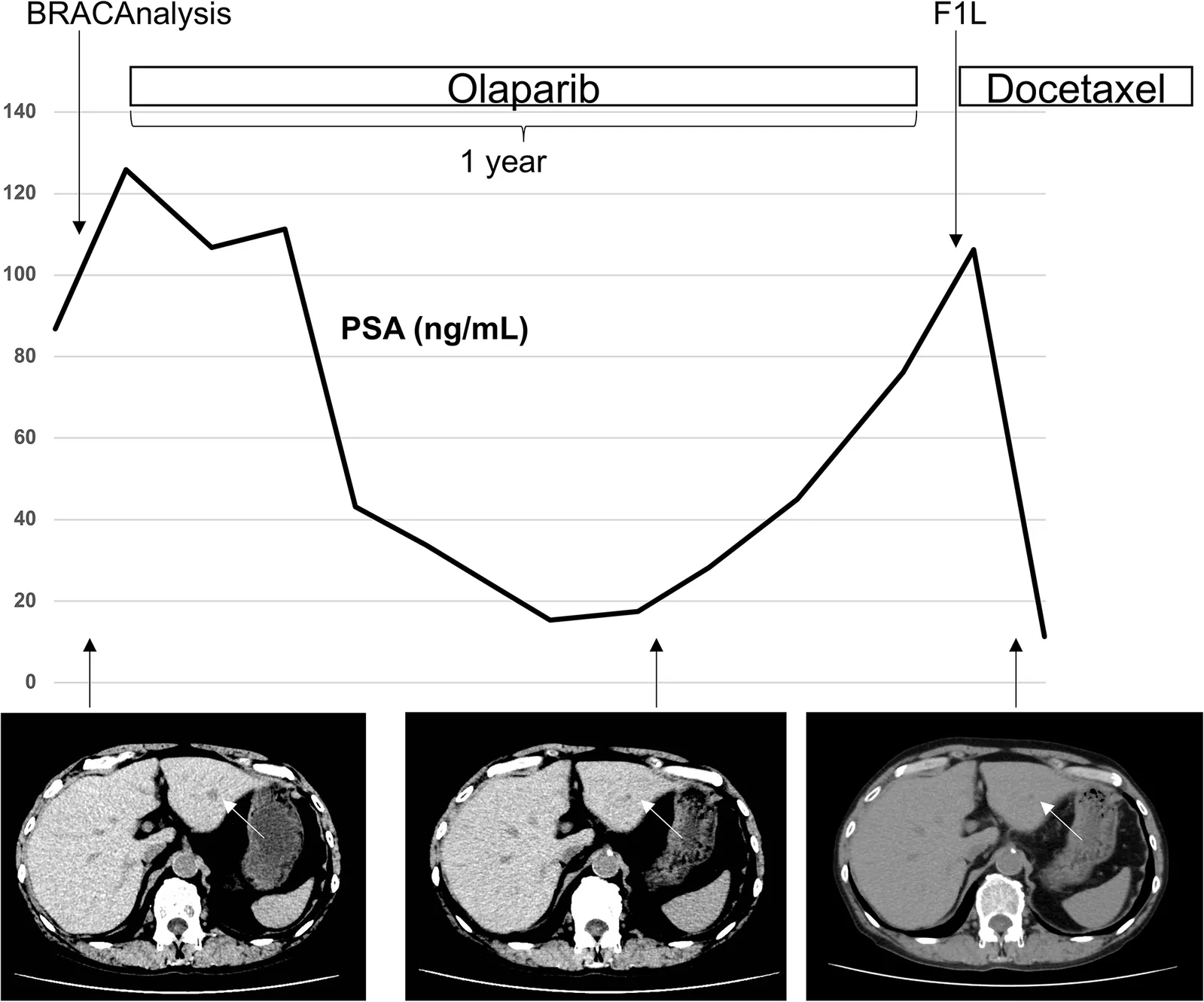
Clinical course of Case 1. Olaparib reduced the PSA level from 125.86 ng/mL to a nadir of 15.40 ng/mL. However, PSA progression was observed one year after initiation. CT images of liver metastases are shown before olaparib initiation, at the time of best PSA response, and after disease progression. Olaparib achieved a partial radiologic response in liver metastases. At the time of PSA progression, no apparent radiological progression was observed in the liver lesions

Treatment with olaparib reduced his PSA from 125.86 to 15.40 ng/mL (nadir), and CT scans showed partial response in liver metastases. The disease remained stable for approximately 1 year before PSA progression was observed (Fig. 1).

Following progression, FoundationOne Liquid CDx^®^, a comprehensive ctDNA-based genomic profiling test, identified 21 additional *BRCA2* mutations (Table 1). Of these, 18 were theoretically capable of restoring the open reading frame disrupted by the germline mutation, consistent with reversion mutations. The proportion of tumor-derived DNA in circulating cell-free DNA was estimated to be 33.3%.

**Table 1:**
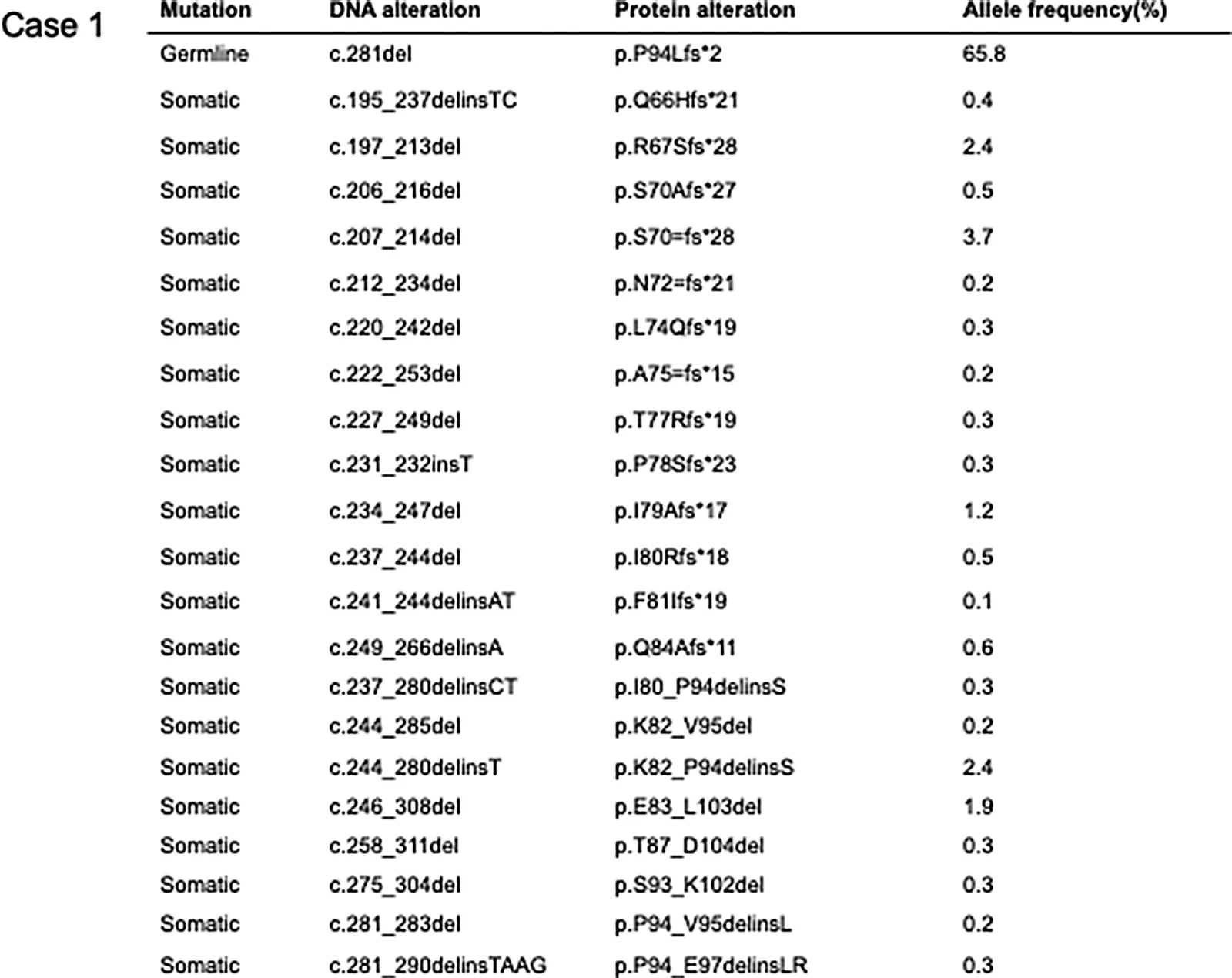
*BRCA2* reversion mutations identified by ctDNA analysis after olaparib resistance in Case 1

FoundationOne Liquid CDx^®^ detected 22 *BRCA2* mutations. Among them, c.281del was the germline mutation also identified by BRACAnalysis^®^, with an allele frequency of 65.8%. The remaining 21 mutations were somatic and undetected by BRACAnalysis^®^, with allele frequencies ranging from 0.2% to 3.7%

## Case 2

A 75-year-old man was diagnosed with mHSPC presenting with multiple liver metastases. His initial PSA level was 46.59 ng/mL, and the prostate biopsy showed Gleason score 5 + 4 = 9 (ISUP Grade Group 5). His family history included breast cancer in his mother and uterine cancer in his sister (Supplement 2).

He received combined androgen blockade (CAB) therapy and IMRT to the prostate (70 Gy/35 fractions). His PSA decreased to a nadir of 1.05 ng/mL but rose again 1 year later. Imaging confirmed disease progression, with worsening liver and right iliac bone metastases.

He was treated with enzalutamide for 6 months, but PSA increased to 17.25 ng/mL. BRACAnalysis^®^ detected a germline *BRCA2* nonsense mutation: c.7558 C > T (p.Arg2520*), introducing a premature stop codon that truncated *BRCA2* and abolished its function.

**Fig. 2:**
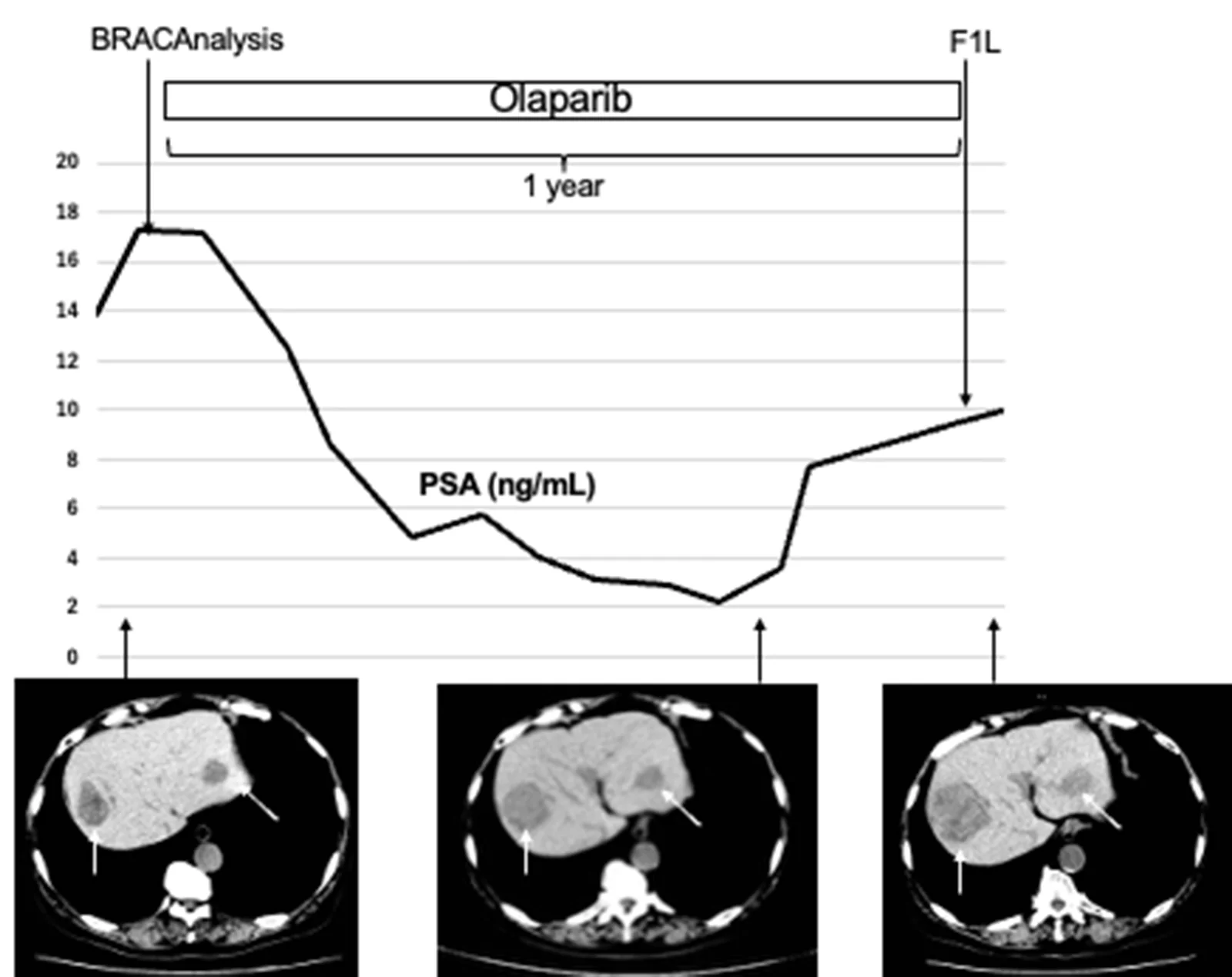
Clinical course of Case 2. Olaparib decreased the PSA level from 17.25 ng/mL to a nadir of 2.18 ng/mL. However, PSA progression was observed one year after initiation. CT scans of liver metastases are shown before olaparib treatment, at the time of best PSA response, and after disease progression. The best radiologic response was stable disease. At the time of PSA progression, radiologic progression of liver metastases was confirmed

Treatment with olaparib reduced PSA from 17.25 to 2.18 ng/mL, with CT scans indicating stable liver disease. This effect was sustained for approximately 1 year (Fig. 2).

Following radiological progression, FoundationOne Liquid CDx^®^ identified a somatic *BRCA2* mutation: c.7558_7559delinsTT (p.Arg2520Leu) (Table 2). This second-site mutation replaced the stop codon introduced by the germline variant with a leucine residue, restoring the open reading frame—consistent with a reversion mutation. The tumor fraction in circulating cell-free DNA could not be determined by the assay.

**Table 2:**
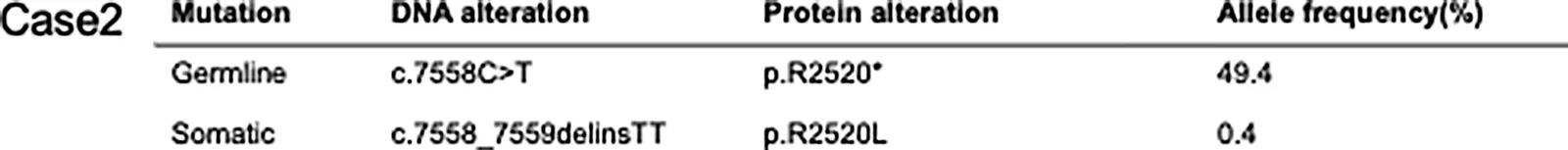
*BRCA2* reversion mutation identified by ctDNA analysis after olaparib resistance in Case 2

FoundationOne Liquid CDx^®^ revealed two *BRCA2* mutations. The germline mutation c.7558 C > T was also identified by BRACAnalysis^®^, with an allele frequency of 49.3%. The somatic reversion mutation c.7558_7559delinsTT was not detected by BRACAnalysis^®^ and had an allele frequency of 0.4%

## Case 3

A 72-year-old man was diagnosed with mHSPC involving lymph node, lung, and bone metastases. His initial PSA was 66.64 ng/mL, and the prostate biopsy showed Gleason score 5 + 5 = 10 (ISUP Grade Group 5). His family history included prostate cancer in his father and breast cancer in his brother (Supplement 3).

He received abiraterone acetate plus prednisolone, resulting in a PSA nadir of 0.04 ng/mL. However, disease progression occurred later, with CT scans revealing worsening bone metastases.

BRACAnalysis^®^ revealed a germline *BRCA2* mutation: c.5851_5854del (p.Ser1951Wfs*11), introducing a premature stop codon 11 bases downstream and disrupting protein function.

**Fig. 3:**
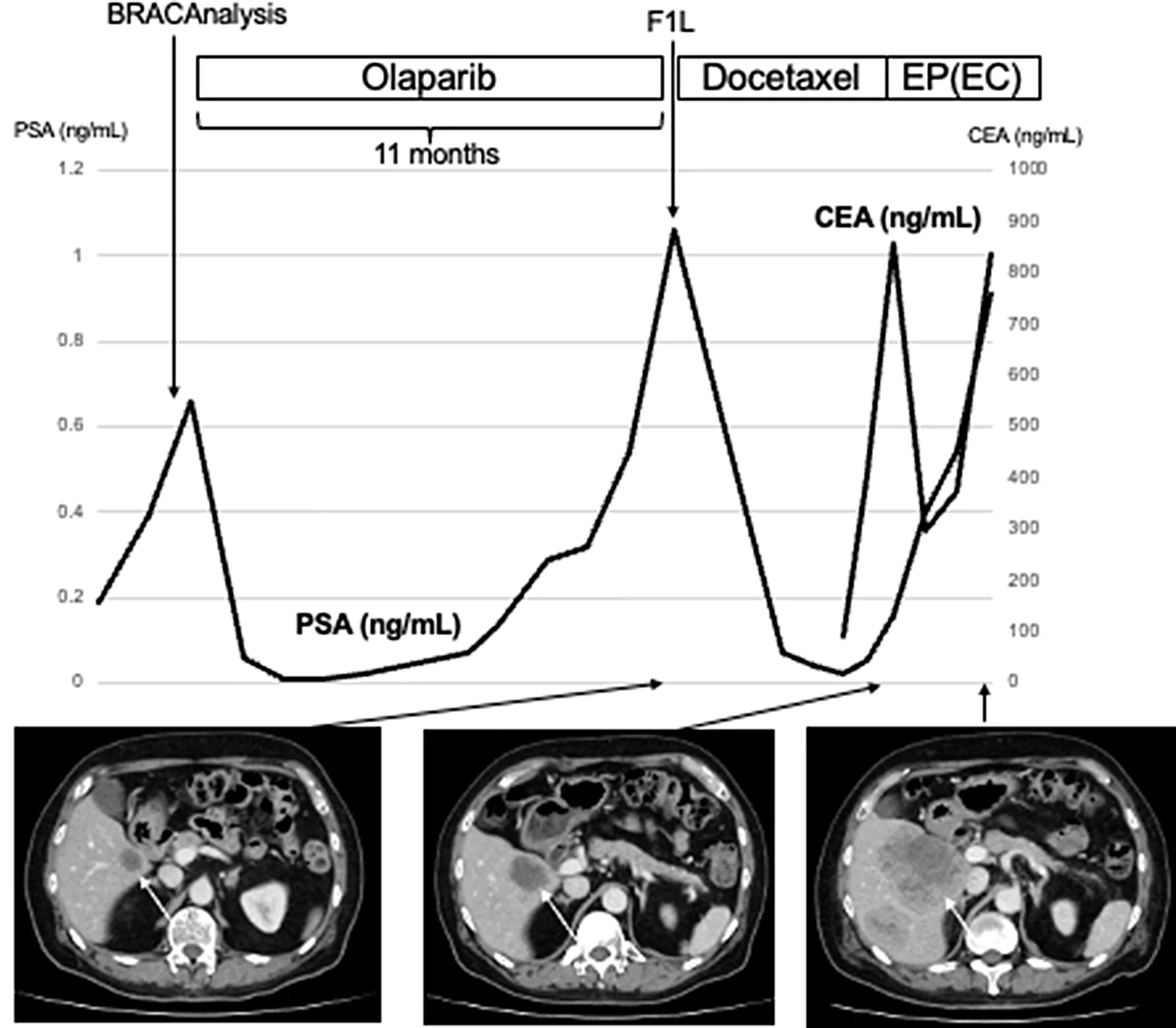
Clinical course of Case 3. Olaparib reduced the PSA level from 0.66 ng/mL to a nadir of 0.01 ng/mL. However, PSA progression occurred 11 months after treatment initiation. CT images of liver metastases are shown after progression on olaparib, after progression on docetaxel plus prednisolone, and after progression on platinum-based chemotherapy (etoposide with cisplatin or carboplatin). Olaparib achieved radiologic stable disease in lymph node, lung, and bone metastases. During docetaxel plus prednisolone treatment, CEA levels increased, and liver biopsy revealed neuroendocrine prostate cancer. Although platinum-based chemotherapy temporarily reduced CEA levels, it did not yield a radiologic response. Disease progression was confirmed after four cycles of the EP (EC) regimen

Treatment with olaparib reduced PSA from 0.66 to 0.01 ng/mL, and CT imaging showed stable disease. This response lasted for 11 months (Fig. 3). Upon progression, with rising PSA (1.06 ng/mL) and new liver metastases, FoundationOne Liquid CDx^®^ identified three somatic *BRCA2* mutations (Table 3). These mutations deleted the region containing the original stop codon, restoring the open reading frame and suggesting reversion. The proportion of tumor-derived DNA in circulating cell-free DNA was estimated to be 48.0%.

**Table 3:**
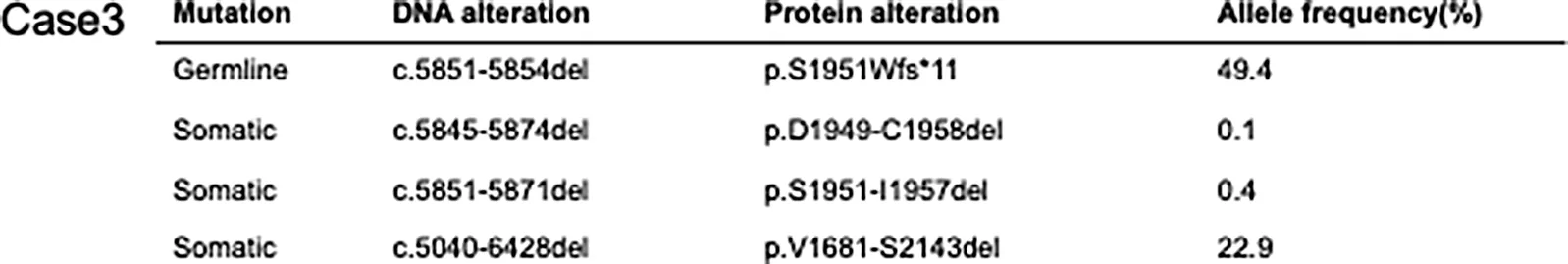
*BRCA2* reversion mutations identified by ctDNA analysis after olaparib resistance in Case 3

FoundationOne Liquid CDx^®^ detected four *BRCA2* mutations. The germline mutation c.5851_5854del was also detected by BRACAnalysis^®^, with an allele frequency of 49.4%. The remaining three somatic mutations were not detected by BRACAnalysis^®^, with allele frequencies ranging from 0.1% to 22.9%

Olaparib was discontinued and replaced with docetaxel plus prednisolone. After 5 cycles, liver metastases progressed. Although PSA declined, carcinoembryonic antigen (CEA) levels increased. A liver biopsy revealed adenocarcinoma with neuroendocrine differentiation, positive for synaptophysin, CD56, and INSM1, and negative for PSA. The patient was diagnosed with neuroendocrine prostate cancer (NEPC).

He was treated with cisplatin for the first cycle, followed by carboplatin for cycles 2–4 due to renal dysfunction, in combination with etoposide. This regimen temporarily reduced CEA levels, but liver metastases progressed again after four cycles.

**Fig. 4:**
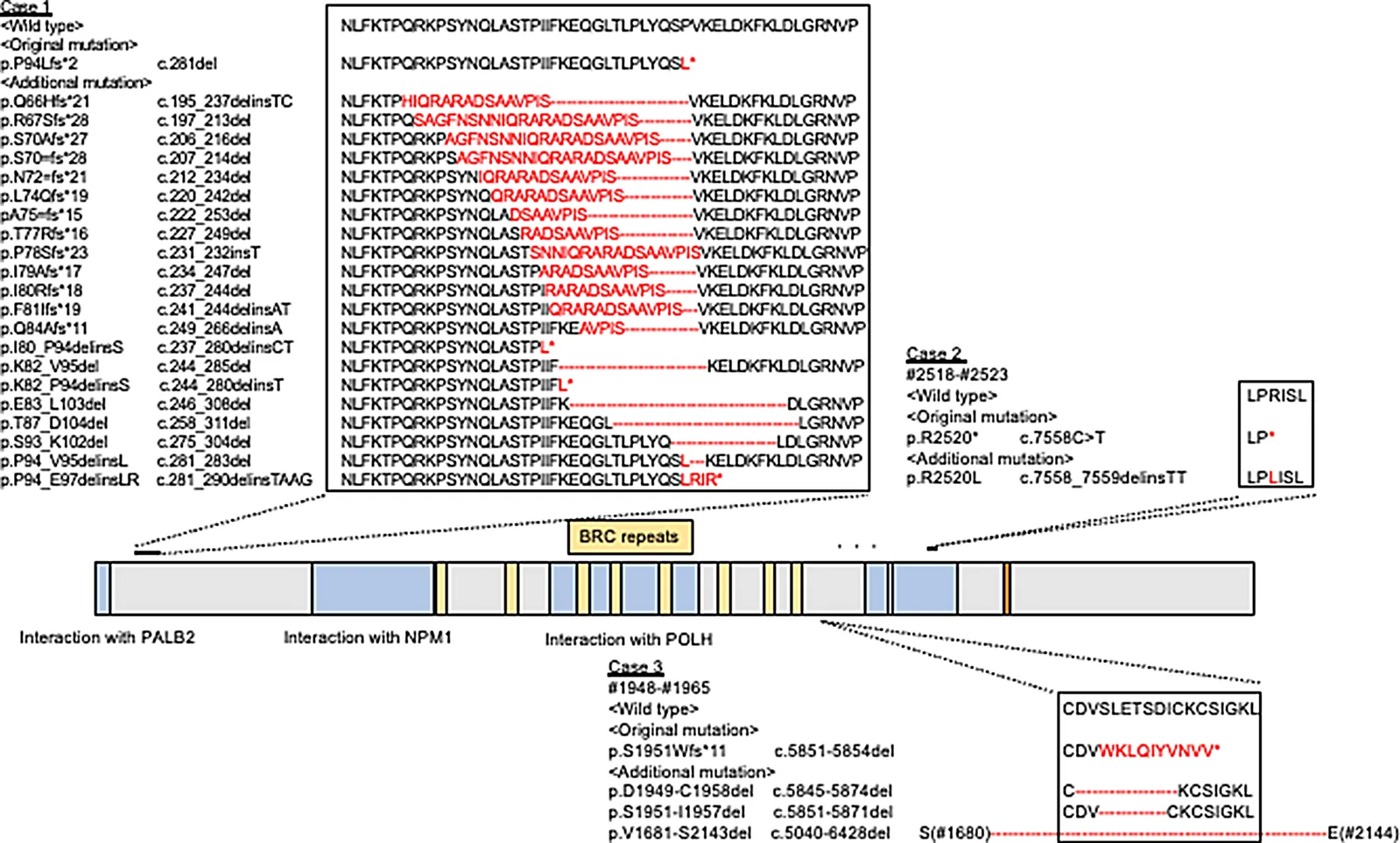
*BRCA2* reversion mutations in Cases 1–3. In Case 1, the original germline mutation was located at codon Pro94. Additional mutations occurred upstream of this site. Of the 21 novel mutations detected by ctDNA analysis, 18 were predicted to restore the open reading frame downstream of the premature stop codon. In Case 2, the original mutation was at codon Arg2520. A somatic mutation at the same position (c.7558_7559delinsTT) replaced the stop codon with leucine, restoring the downstream amino acid sequence. In Case 3, the germline mutation was at codon Ser1951. Additional mutations occurred at or upstream of the same site, and all three somatic mutations restored the open reading frame downstream of the original stop codon

The identified BRCA2 reversion mutations and their predicted effects on the open reading frame in Cases 1–3 are summarized in Fig. 4.

## Discussion

*BRCA1* and *BRCA2* are tumor suppressor genes that play essential roles in homologous recombination–mediated repair of DNA double-strand breaks [5]. Poly(ADP-ribose) polymerase (PARP) is crucial for the repair of both single- and double-strand DNA breaks, and its inhibition leads to synthetic lethality in cancer cells with defective BRCA function [6]. PARP inhibitors such as olaparib and rucaparib have demonstrated clinical efficacy in *BRCA*-altered patients with mCRPC in phase 3 clinical trials [7, 8]. As a result, olaparib has become a standard treatment option for mCRPC patients with either germline or somatic *BRCA1/2* mutations. Nevertheless, resistance to PARP inhibitors remains a significant clinical challenge.

In this report, we describe three patients with mCRPC and germline *BRCA2* mutations who initially responded to olaparib but subsequently developed resistance. In each case, additional *BRCA2* mutations were identified at the time of disease progression using circulating tumor DNA (ctDNA) analysis via FoundationOne Liquid CDx^®^. In Case 1, the germline c.281del mutation caused a frameshift and premature stop codon, and 21 additional *BRCA2* mutations were detected after progression, 18 of which were predicted to restore the open reading frame. In Case 2, a nonsense mutation (c.7558C > T) was reversed by a somatic mutation (c.7558_7559delinsTT), which replaced the stop codon with a leucine residue. In Case 3, somatic deletions removed the region containing the original premature stop codon (c.5851_5854del), suggesting restoration of BRCA2 function. These observations suggest that *BRCA2* reversion mutations likely contributed to acquired resistance to olaparib in all three patients.

This case series has several limitations. First, we did not perform functional validation to confirm whether the secondary mutations restored BRCA2 protein function. Second, the phasing of somatic mutations relative to the germline variant remains unknown, and the reversion hypothesis assumes that these mutations occur in cis. Third, since BRACAnalysis^®^ utilizes DNA from peripheral blood mononuclear cells, we were unable to determine whether the reversion mutations were acquired during treatment or present at low frequency beforehand. Nonetheless, the fact that reversion mutations were detected at the time of clinical progression and not before treatment initiation supports their role in acquired resistance. In addition, the estimated tumor fractions in circulating cell-free DNA were 33.3% in Case 1 and 48.0% in Case 3, supporting the reliability of ctDNA-based variant detection.

Several mechanisms of resistance to PARP inhibitors have been described, including upregulation of P-glycoprotein, which facilitates drug efflux [9], loss of 53BP1, which restores homologous recombination [10], and *BRCA2* reversion mutations, which correct the disrupted open reading frame and restore protein function [3, 4]. In ovarian cancer, such reversion mutations have been associated with resistance to both PARP inhibitors and platinum-based chemotherapy [11].

*BRCA2* reversion mutations have been recognized as an important mechanism of acquired resistance to PARP inhibitors in prostate cancer. Translational analyses from the TOPARP-A trial demonstrated the emergence of multiple subclonal *BRCA2* and *PALB2* reversion mutations detected by serial ctDNA analysis at the time of disease progression following olaparib treatment [12]. Additional clinical reports and translational studies have further supported the role of *BRCA2* reversion mutations in PARP inhibitor resistance in mCRPC [13–16]. More recently, comprehensive longitudinal ctDNA analyses from the TOPARP-B trial demonstrated that *BRCA2/PALB2* reversion mutations emerged in 79% of tumors at the end of treatment, often with multiple resistant subclones developing in parallel over time [17]. Similar to the findings from TOPARP-A, Case 1 in our series demonstrated the emergence of multiple putative *BRCA2* reversion mutations, suggesting heterogeneous resistant subclones under treatment-selective pressure.

The number of reversion events in previous reports varied from 1 to 17, despite similar clinical courses and treatment durations. In our series, the number of reversion mutations ranged from 1 to 18. This variability may reflect differences in the position and biological impact of the original germline mutation, the extent of exposure to PARP inhibitors, or tumor heterogeneity and selective pressure during treatment. In the TRITON2 trial, ctDNA from 100 patients with mCRPC treated with rucaparib was evaluated, and 39% developed *BRCA* reversion mutations following treatment [18]. Among those, 74% exhibited two or more reversion mutations, with a longer treatment duration and better clinical response associated with a greater number of reversions.

In Japan, *BRCA* testing in prostate cancer can be performed using BRACAnalysis^®^, which detects germline mutations from peripheral blood but cannot identify somatic or reversion mutations. FoundationOne CDx^®^ (tissue) and FoundationOne Liquid CDx^®^ (ctDNA) are capable of detecting both somatic and germline mutations, including reversions. However, their use is limited by insurance coverage, which typically permits testing only once and only after standard therapies have failed. Tissue-based tests may not capture tumor heterogeneity, while liquid biopsies can provide a more comprehensive view of the tumor’s genetic profile, making them preferable for identifying reversion mutations at the time of progression.

We recommend a stepwise approach to *BRCA* testing in clinical practice. BRACAnalysis^®^ can be used at diagnosis to assess eligibility for olaparib. At the time of disease progression, FoundationOne Liquid CDx^®^ can be employed to investigate the presence of resistance mechanisms, including reversion mutations. The PROfound study demonstrated high concordance between tissue and ctDNA testing for *BRCA* and *ATM* mutations, with positive agreement of 81% and negative agreement of 92%, particularly high for nonsense (93%), splice-site (87%), and frameshift (86%) variants [19]. These findings support the reliability of ctDNA-based testing for guiding clinical decision-making, especially in cases where tumor tissue is unavailable or outdated.

## Conclusion

We report three cases of *BRCA2* reversion mutations detected by ctDNA analysis in mCRPC patients who developed resistance to olaparib after initial clinical benefit.

These reversion mutations likely contributed to the loss of PARP inhibitor efficacy and represent an important resistance mechanism. Detecting such mutations is crucial for guiding treatment changes.

Understanding the characteristics and limitations of each genetic test allows clinicians to make informed decisions and optimize therapy. Incorporating liquid biopsy into routine practice can improve the detection of clinically relevant mutations, including those associated with therapeutic resistance.

## Supplementary Information

Below is the link to the electronic supplementary material.

Supplementary Material 1: Supplement 1. Family history in Case 1. The patient in Case 1 had a family history of cancer, including a father diagnosed with colorectal cancer at the age of 83 and a maternal aunt with gastric cancer (age at diagnosis unknown).

Supplementary Material 2: Supplement 2. Family history in Case 2. The patient in Case 2 had a family history of cancer, including a mother with breast cancer and a sister with uterine cancer, both of whom died in their 80s and 40s, respectively (ages at diagnosis unknown).

Supplementary Material 3: Supplement 3. Family history in Case 3. The patient in Case 3 had a family history of cancer, including a father diagnosed with prostate cancer at the age of 80 years and a brother diagnosed with breast cancer in his 70s and leukemia at the age of 70 years.

## Data Availability

All data generated or analyzed during this study are included in this published article and its supplementary information files. Additional datasets are available from the corresponding author upon reasonable request.

## Abbreviations

BRCA1/2: Breast cancer susceptibility gene 1 and 2
PARP: Poly(ADP-ribose) polymerase
ctDNA: Circulating tumor DNA
mCRPC: Metastatic castration-resistant prostate cancer
mHSPC: Metastatic hormone-sensitive prostate cancer
PSA: Prostate-specific antigen
IMRT: Intensity-modulated radiation therapy
CAB: Combined androgen blockade
CEA: Carcinoembryonic antigen
NEPC: Neuroendocrine prostate cancer
HR: Homologous recombination
HRR: Homologous recombination repair
CDx: Companion diagnostic
PALB2: Partner and localizer of BRCA2
